# Recipient Comorbidities for Prediction of Primary Graft Dysfunction, Chronic Allograft Dysfunction and Survival After Lung Transplantation

**DOI:** 10.3389/ti.2022.10451

**Published:** 2022-06-29

**Authors:** Jonas Peter Ehrsam, Macé M. Schuurmans, Mirjam Laager, Isabelle Opitz, Ilhan Inci

**Affiliations:** ^1^ Department of Thoracic Surgery, University Hospital Zurich, Zurich, Switzerland; ^2^ Division of Pulmonology, University Hospital Zurich, Zurich, Switzerland; ^3^ Department of Biostatistics, University of Basel, Basel, Switzerland

**Keywords:** lung transplantation, primary graft dysfunction, recipient selection, comorbidities, Charlson-Deyo-Index, chronic allograft dysfunction

## Abstract

Since candidates with comorbidities are increasingly referred for lung transplantation, knowledge about comorbidities and their cumulative effect on outcomes is scarce. We retrospectively collected pretransplant comorbidities of all 513 adult recipients transplanted at our center between 1992–2019. Multiple logistic- and Cox regression models, adjusted for donor-, pre- and peri-operative variables, were used to detect independent risk factors for primary graft dysfunction grade-3 at 72 h (PGD3-T72), onset of chronic allograft dysfunction grade-3 (CLAD-3) and survival. An increasing comorbidity burden measured by Charleston-Deyo-Index was a multivariable risk for survival and PGD3-T72, but not for CLAD-3. Among comorbidities, congestive right heart failure or a mean pulmonary artery pressure >25 mmHg were independent risk factors for PGD3-T72 and survival, and a borderline risk for CLAD-3. Left heart failure, chronic atrial fibrillation, arterial hypertension, moderate liver disease, peptic ulcer disease, gastroesophageal reflux, diabetes with end organ damage, moderate to severe renal disease, osteoporosis, and diverticulosis were also independent risk factors for survival. For PGD3-T72, a BMI>30 kg/m2 was an additional independent risk. Epilepsy and a smoking history of the recipient of >20packyears are additional independent risk factors for CLAD-3. The comorbidity profile should therefore be closely considered for further clinical decision making in candidate selection.

## Graphical Abstract

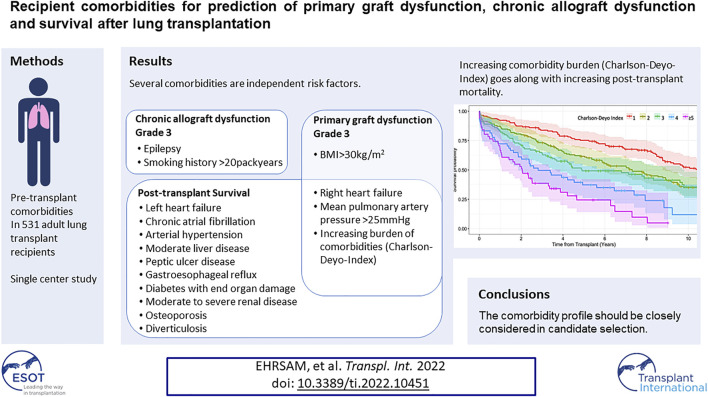

## Introduction

Comorbidities in lung transplant candidates have increasingly been accepted over the last decades in parallel with steadily increasing numbers of lung transplantation procedures over time. This broadening of acceptable candidates was partly supported by the International Society for Heart and Lung Transplantation (ISHLT) consensus report for the selection of lung transplant candidates, published in 1998 and updated in 2006, 2014 (1) and 2021 (2). However, these consensus reports are based mainly on expert opinion. Strong evidence about comorbidities and their impact on primary graft dysfunction (PGD), chronic allograft dysfunction (CLAD), and survival are still missing. Moreover, almost nothing is known about the cumulative effect of comorbidities in a potential lung transplant candidate. In other fields of medicine, the cumulative effect of comorbidities for prognostic assessment has been extensively studied. One of the most commonly used comorbidity models is the Charlson-Comorbidity-Index introduced in 1987 (3). This index is based on comorbid conditions with varying assigned weights, resulting in a composite score. As increasing age was shown to be more an expression of accumulation of comorbidities than an actual risk factor per se, an age independent version, the Charlson-Deyo-Index (CDI)(4) was proposed. Among transplant patients, the CDI and its derivates has shown to be predictive in recipients undergoing renal transplantation (5, 6) and liver transplantation (7, 8).

In the era of organ shortage, it is of paramount importance to know which patient and at which time point will benefit from lung transplantation for an extended time period. We investigated the impact of a large variety of pretransplant comorbidities among our recipients transplanted at our center in respect to PGD, CLAD and survival. For cumulative comorbidity conditions, we additionally evaluated the CDI for the same outcomes.

## Methods

We systematically, retrospectively collected data from medical records of all adult recipients and their corresponding donors transplanted at the University Hospital of Zurich between 11/1992 and 12/2019, with last follow-up in 01/2022. Recipient selection was based on a liberal use of the updated ISHLT consensus document (1). All comorbidity variables were based on the most immediate pretransplantation data. Follow-up of the recipients was performed in our outpatient department or in close quarterly to half-yearly exchange with other institutions.

### Definition of the Charlson-Deyo-Index

This index (4) is age independent and estimates the impact of multiple comorbidities. It considers 19 comorbid conditions (ranging from 1 to 6 points), of which 1 point was always reserved by the chronic pulmonary disease in each of our recipients. All included comorbidities and their assigned points are listed in Tables 1–3. An increasing score of points represents an increasing category of risk.

**TABLE 1:** Pre-transplant recipient characteristics for survival.

	N = 513	Univariable analysis			Multivariable analysis			
		HR	95% CI	*p*	Model	HR	95% CI	*p*
Recipient Characteristics								
Age (median, range)	49 (18–70)	1.02	1.02–1.03	0.000	A, B, C, D	1.01	1.00–1.02	0.004
Sex male	270 (52.6%)	1.14	0.92–1.41	0.220				
Diagnosis								
Cystic fibrosis	156 (30.4%)	0.57	0.45–0.73	0.000				
Idiopathic pulmonary arterial hypertension	27 (5.2%)	1.33	0.86–2.07	0.205				
Emphysema	155 (30.2%)	1.19	0.95–1.49	0.133				
Idiopathic pulmonary fibrosis	111 (21.6%)	1.54	1.21–1.97	0.001				
Other	64 (12.5%)							
Smoking (pack years) (median, range)	0 (0–120)	1.01	1.00–1.01	0.006				
>20py	187 (36.5%)	1.34	1.08–1.66	0.009				
Waitlist (days) (median, range)	150 (0–1965)	1.00	1.00–1.00	0.525				
Recipient Comorbidities								
Any coronary artery disease	58 (11.3%)	1.71	1.23–2.37	0.001				
Myocardial infarction^a^(1pt)	7 (1.4%)	2.44	1.01–5.93	0.048				
Postinterventional coronary disease (stent)	16 (3.1)	1.47	0.82–2.61	0.194				
Coronary disease mild	43 (8.4%)	1.68	1.16–2.44	0.006				
Congestive heart failure^a^(1pt)	267 (52.0%)	2.13	1.71–2.64	0.000	A	1.91	1.53–2.40	0.004
Right heart failure	262 (51.1%)	2.04	1.65–2.53	0.000	C	1.81	1.45–2.28	0.000
mPAP (median, range)	28 (17–82)	1.02	1.01–1.03	0.000	B, C	1.64	1.31–2.06	0.000
>25 mmHg	264 (51.5%)	1.91	1.54–2.37	0.000				
Left heart failure	12 (2.3%)	3.62	1.97–6.64	0.000	C	2.07	1.11–3.87	0.023
Chronic atrial fibrillation	26 (5.1%)	3.33	2.10–5.29	0.000	B	2.10	1.31–3.38	0.002
Systemic hypertension	138 (26.9%)	2.02	1.60–2.56	0.000	B, C	1.33	1.03–1.72	0.028
Peripheral vascular disease^a^(1pt)	18 (3.5%)	1.86	1.06–3.25	0.030				
Peripheral artery disease grade I	12 (2.3%)	1.24	0.58–2.62	0.579				
Aortic dissection	3 (0.6%)	5.82	1.86–18.26	0.003				
Aortic ectasia	4 (0.8%)	2.92	1.08–7.86	0.034				
Cerebrovascular disease^a^(1pt)	11 (2.1%)	0.97	0.46–2.04	0.927				
Hemiplegia^a^(2pt)	0							
Epilepsy	6 (1.2%)	1.08	0.45–2.61	0.866				
Dementia^a^(1pt)	0							
Connecstive tissue disease^a^(1pt)	22 (4.3)	0.89	0.52–1.53	0.683				
Rheumatoid arthritis	10 (1.9%)	1.66	0.82–3.36	0.156				
Scleroderma	6 (1.2%)	0.44	0.14–1.39	0.163				
Liver disease mild^a^(1pt)	78 (15.2%)	1.17	0.85–1.60	0.350				
Liver disease moderate^a^(3pt)	12 (2.3%)	1.49	1.19–1.87	0.000	A, B, C	1.41	1.12–1.77	0.004
Peptic ulcer disease^a^(1pt)	18 (3.5%)	2.49	1.48–4.19	0.001	A, B, C	1.78	1.00–3.24	0.040
Gastroesophageal reflux	147 (28.7%)	1.67	1.32–2.12	0.000	A, B, C	1.28	1.00–1.65	0.023
Barret oesophagus	17 (3.3%)	1.44	0.81–2.57	0.217				
Chronic pulmonary disease^a^(1pt)	513 (100.0%)							
Diabetes mild^a^(1pt)	90 (17.5%)	0.85	0.64–1.13	0.262				
Diabetes end-organ damage^a^(2pt)	8 (1.6%)	1.45	1.01–2.07	0.043	A, B, C	1.59	1.11–2.28	0.012
Moderate or severe renal disease^a^(2pt)	61 (11.9%)	1.64	1.41–1.92	0.000	A, B, C	1.38	1.18–1.62	0.000
BMI (median, range)	20.8 (13.1–38.1)	1.05	1.03–1.07	0.000				
30.0–34.9	28 (5.5%)	1.42	0.92–2.19	0.112				
≥35	4 (0.8%)	3.09	1.15–8.31	0.025				
<18.5	142 (27.7%)	0.77	0.61–0.98	0.031				
Osteoporosis	178 (34.7%)	1.52	1.22–1.89	0.000	A, B, C	1.52	1.21–1.92	0.000
Diverticulosis	65 (12.7%)	2.02	1.48–2.75	0.000	A, B, C	1.42	1.01–2.00	0.043
Morbus Crohn/Colitis ulcerosa	6 (1.2%)	1.21	0.39–3.78	0.743				
Cholecystolithiasis	30 (5.8%)	1.23	0.78–1.93	0.373				
Pre-transplant critical situation (e.g., MV, ECMO, ICU)	56 (10.9%)	1.53	1.08–2.17	0.017				
Pre-transplant ECMO	34 (6.6%)	1.51	0.97–2.35	0.071				
Lymphoma^a^(2pt)	6 (1.2%)	0.75	0.43–1.33	0.331				
Leukemia^a^(2pt)	1 (0.2%)	2.38	0.89–6.38	0.085				
Tumor^a^(2pt)	24 (4.7%)	1.18	0.92–1.50	0.198				
Metastatic solid tumor^a^(6pt)	0							
AIDS^a^(6pt)	0							
^a^Charlson-Deyo-Index pt (median, range)	2 (1–8)	1.37	1.26–1.48	0.000				
1	142 (27.7%)				D	Ref		
2	166 (32.4%)					1.56	1.18–2.05	0.002
3	100 (19.5%)					1.65	1.19–2.30	0.003
4	54 (10.5%)					3.08	2.11–4.50	0.000
≥5	51 (9.9%)					4.10	2.76–6.09	0.000
Transplant and Donor Characteristics								
Era 1992–2000 vs. 2001–2019	98 (19.1%)	1.31	1.00–1.71	0.051				
Era 1992–2008 vs. 2009–2019	247 (48.1%)	1.22	0.97–1.54	0.093				
Unilateral Transplantation	36 (7.0%)	2,01	1.41–2.87	0.000	A, B, C, D	2.68	1.85–3.87	0.000
Re-Transplantation	23 (4.5%)	2.41	1.53–3.80	0.000				
Intra-operative ECMO use	241 (47.0%)	1.40	1.14–1.73	0.002				
CMV high risk	131 (25.5%)	1.01	0.79–1.28	0.961				
Zurich Donor Score, median (range)	3 (0–12)	1.13	1.09–1.18	0.000	A, B, C, D	1.10	1.06–1.15	0.000
DCD	28 (5.5%)	0.90	0.50–1.61	0.718				
EVLP	10 (1.9%)	0.78	0.32–1.88	0.575				
PGD3 at T72	79 (15.4%)	2.07	1.58–2.70	0.000				

a Variables and points (pt) of Charlson-Deyo-Index.

Abbreviations: AIDS, acquired immune deficiency syndrome; BMI, body mass index; CI, confidence interval; CMV, cytomegalo virus; DCD, lung donation after circulatory death; HR, hazard ratio; MV, mechanical ventilation; ECMO, extracorporeal membrane oxygenation; EVLP, *ex vivo* lung perfusion; FEV1, forced expiratory volume in 1 s; ICU, intensive care unit; mPAP, mean pulmonary artery pressure; OR, odds ratio; PGD, primary graft dysfunction py, pack years.

**TABLE 2:** Pre-transplant recipient characteristics for PGD3 on day 3.

	N = 79/507	Univariable analysis			Multivariable analysis			
		OR	95% CI	*p*	Model	OR	95% CI	*p*
Recipient Characteristics								
Age (median, range)	48 (18–68)	1.01	0.99–1.02	0.586				
Sex male	38 (48.1%)	0.81	0.50–1.31	0.398				
Diagnosis								
Cystic fibrosis	18 (22.8%)	0.62	0.35–1.09	0.096				
Idiopathic pulmonary arterial hypertension	14 (17.7%)	6.36	0.29–13.97	0.000				
Emphysema	8 (10.1%)	0.22	0.10–0.46	0.000				
Idiopathic pulmonary fibrosis	28 (35.4%)	2.35	1.40–3.96	0.001				
Other	18 (22.8%)							
Smoking (pack years) (median, range)	0 (0–80)	0.98	0.97–1.00	0.015				
>20py	21 (26.6%)	0.58	0.34–1.00	0.048				
Waitlist (days) (median, range)	39 (11–88)	1.00	1.00–1.00	0.274				
Recipient Comorbidities								
Any coronary artery disease	5 (6.3%)	0.48	0.19–1.24	0.128				
Myocardial infarction^a^(1pt)	1 (1.3%)	0.90	0.11–7.59	0.924				
Postinterventional coronary disease (stent)	2 (2.5%)	0.83	0.18–3.75	0.808				
Coronary disease mild	3 (3.8%)	0.38	0.12–1.27	0.117				
Congestive heart failure^a^(1pt)	64 (81.0%)	5.00	2.76–9.06	0.000	A	4.28	2.34–7.83	0.000
Right heart failure	63 (79.7%)	4.79	2.68–8.57	0.000	C	2.47	1.28–4.80	0.007
mPAP (median, range)	35 (20–80)	1.04	1.03–1.06	0.000	B	2.15	1.12–4.15	0.022
>25 mmHg	62 (78.5%)	4.32	2.44–7.62	0.000				
Left heart failure	4 (5.1%)	3.21	0.92–11.23	0.068				
Chronic atrial fibrillation	6 (7.6%)	1.87	0.72–4.87	0.199				
Systemic hypertension	25 (31.6%)	1.31	0.78–2.20	0.315				
Peripheral vascular disease^a^(1pt)	2 (2.5%)	0.67	0.15–2.97	0.597				
Peripheral artery disease grade I	0	—						
Aortic dissection	1 (1.3%)	2.73	0.25–30.48	0.414				
Aortic ectasia	1 (1.3%)	1.82	0.19–17.69	0.607				
Cerebrovascular disease^a^(1pt)	0	—						
Hemiplegia^a^(2pt)	0	—						
Epilepsy	0	—						
Dementia^a^(1pt)	0	—						
Connective tissue disease^a^(1pt)	7 (8.9%)	2.68	1.06–6.79	0.038				
Rheumatoid arthritis	3 (3.8%)	2.37	0.60–9.38	0.218				
Scleroderma	3 (3.8%)	5.59	1.11–28.22	0.037				
Liver disease mild^a^(1pt)	14 (17.7%)	1.25	0.66–2.36	0.495				
Liver disease moderate^a^(3pt)	2 (2.5%)	1.07	0.64–1.79	0.810				
Peptic ulcer disease^a^(1pt)	1 (1.3%)	0.31	0.04–2.36	0.258				
Gastroesophageal reflux	22 (27.8%)	0.99	0.58–1.69	0.973				
Barret oesophagus	0	—						
Chronic pulmonary disease^a^(1pt)	79 (100.0%)	—						
Diabetes mild^a^(1pt)	12 (15.2%)	0.83	0.43–1.61	0.580				
Diabetes end-organ damage^a^(2pt)	2 (2.5%)	1.48	0.65–3.40	0.352				
Moderate or severe renal disease^a^(2pt)	11 (13.9%)	1.12	0.79–1.59	0.532				
BMI (median, range)	22.8 (14.7–36.0)	1.09	1.04–1.15	0.001				
≥30.0	13 (16.5%)	5.42	2.47–11.91	0.000	A, B, C, D	4.27	1.88–9.68	0.001
≥35	2 (2.5%)	5.53	0.77–39.87	0.090				
<18.5	19 (24.1%)	0.80	0.46–1.40	0.441				
Osteoporosis	33 (41.8%)	1.43	0.88–2.33	0.153				
Diverticulosis	15 (19.0%)	1.81	0.96–3.43	0.067				
Morbus Crohn/Colitis ulcerosa	0	—						
Cholecystolithiasis	3 (3.8%)	0.59	0.17–1.98	0.390				
Pre-transplant critical situation (e.g., MV, ECMO, ICU)	14 (17.7%)	2.15	1.11–4.18	0.024				
Pre-transplant ECMO	10 (12.7%)	2.81	1.27–6.22	0.011				
Lymphoma^a^(2pt)	1 (1.3%)	1.04	0.35–3.07	0.941				
Leukemia^a^(2pt)	0	—						
Tumor^a^(2pt)	3 (3.8%)	0.90	0.48–1.67	0.732				
Metastatic solid tumor^a^(6pt)	0	—						
AIDS^a^(6pt)	0	—						
^a^Charlson-Deyo-Index pt (median, range)	2 (1–6)	1.22	1.04–1.45	0.017				
1					D	Ref		
2						3.42	1.54–7.57	0.002
3						2.45	1.01–5.91	0.047
≥4						3.75	1.60–8.77	0.002
Transplant and Donor Characteristics								
Era 1992–2000 vs. 2001–2019	11 (13.9%)	1.58	0.80–3.11	0.188				
Era 1992–2008 vs. 2009–2019	44 (55.7%)	0.71	0.44–1.15	0.166				
Unilateral Transplantation	3 (3.8%)	0.49	0.15–1.64	0.245				
Re-Transplantation	1 (1.3%)	0.26	0.04–1.98	0.194				
Intra-operative ECMO use	60 (75.9%)	1.52	2.61–7.84	0.000	A, B, C	2.93	1.56–5.53	0.001
CMV high risk	17 (21.5%)	0.76	0.42–1.35	0.341				
Zurich Donor Score, median (range)	3 (0–11)	1.14	1.04–1.24	0.003	A, B, C, D	1.11	1.01–1.21	0.028
DCD	3 (3.8%)	0.64	0.19–2.16	0.469				
EVLP	2 (2.5%)	0.73	0.15–3.52	0.698				

a Variables and points (pt) of Charlson-Deyo-Index.

Abbreviations: AIDS, acquired immune deficiency syndrome; BMI, body mass index; CI, confidence interval; CMV, cytomegalo virus; DCD, lung donation after circulatory death; HR, hazard ratio; MV, mechanical ventilation; ECMO, extracorporeal membrane oxygenation; EVLP, *ex vivo* lung perfusion; FEV1, forced expiratory volume in 1 s; ICU, intensive care unit; mPAP, mean pulmonary artery pressure; OR, odds ratio; PGD, primary graft dysfunction py, pack years.

**TABLE 3:** Pre-transplant recipient characteristics for onset of CLAD-3.

	N = 266/513	Univariable analysis			Multivariable analysis			
		HR	95% CI	*p*	Model	HR	95% CI	*p*
Recipient Characteristics								
Age (median, range)	51 (18–70)	1.01	1.00–1.02	0.002				
Sex male	143 (53.8%)	1.11	0.88–1.41	0.380				
Diagnosis								
Cystic fibrosis	69 (25.9%)	0.69	0.53–0.91	0.007				
Idiopathic pulmonary arterial hypertension	13 (4.9%)	0.82	0.48–1.40	0.470				
Emphysema	86 (32.3%)	1.17	0.91–1.50	0.230				
Idiopathic pulmonary fibrosis	64 (24.1%)	1.38	1.03–1.86	0.031	A, B, D	1.44	1.07–1.95	0.017
Other								
Smoking (pack years) (median, range)	4 (0–120)	1.01	1.00–1.01	0.001	A, B, C, D	1.48	1.16–1.91	0.002
>20py	112 (42.1%)							
Waitlist (days) (median, range)	150.5 (0–1378)	1.00	1.00–1.00	0.820				
Recipient Comorbidities								
Any coronary artery disease	32 (12.0%)	1.33	0.89–1.98	0.160				
Myocardial infarction^a^(1pt)	2 (0.8%)	0.57	0.12–2.66	0.470				
Postinterventional coronary disease (stent)	8 (3.0%)	0.95	0.41–2.18	0.900				
Coronary disease mild	24 (9.0%)	1.48	0.95–2.30	0.080				
Congestive heart failure^a^(1pt)	142 (53.4%)	1.30	1.03–1.64	0.030	A	1.27	1.00–1.16	0.053
Right heart failure	140 (52.6%)	1.31	1.04–1.66	0.023	B	1.24	0.98–1.58	0.078
mPAP (median, range)	32 (20–80)	1.01	1.00–1.02	0.038	C	1.23	0.97–1.57	0.092
>25 mmHg	141 (53.0%)							
Left heart failure	6 (2.3%)	1.10	0.41–2.93	0.850				
Chronic atrial fibrillation	9 (3.4%)	0.67	0.33–1.38	0.280				
Systemic hypertension	74 (27.8%)	1.29	0.97–1.70	0.077				
Peripheral vascular disease^a^(1pt)	5 (1.9%)	0.50	0.20–1.27	0.140				
Peripheral artery disease grade I	2 (0.8%)	0.29	0.07–1.19	0.085				
Aortic dissection	1 (0.4%)	0.61	0.07–5.50	0.660				
Aortic ectasia	3 (1.1%)	2.13	0.62–7.27	0.230				
Cerebrovascular disease^a^(1pt)	6 (2.3%)	1.32	0.62–2.81	0.460				
Hemiplegia^a^(2pt)	0							
Epilepsy	5 (1.9%)	1.92	0.90–4.07	0.089	A, B, C, D	2.34	1.06–5.19	0.036
Dementia^a^(1pt)	0							
Connective tissue disease^a^(1pt)	15 (5.6%)	1.44	0.87–2.39	0.150				
Rheumatoid arthritis	8 (3.0%)	2.24	1.07–4.72	0.033				
Scleroderma	4 (1.5%)	1.10	0.46–2.67	0.830				
Liver disease mild^a^(1pt)	29 (10.9%)	0.74	0.49–1.10	0.130				
Liver disease moderate^a^(3pt)	4 (1.5%)	0.87	0.62–1.22	0.410				
Peptic ulcer disease^a^(1pt)	6 (2.3%)	0.73	0.28–1.85	0.500				
Gastroesophageal reflux	71 (26.7%)	1.05	0.80–1.38	0.740				
Barret oesophagus	9 (3.4%)	1.10	0.57–2.12	0.780				
Chronic pulmonary disease^a^(1pt)	266 (100.0%)							
Diabetes mild^a^(1pt)	41 (15.4%)	0.82	0.59–1.13	0.230				
Diabetes end-organ damage^a^(2pt)	5 (1.9%)	1.17	0.72–1.90	0.520				
Moderate or severe renal disease^a^(2pt)	25 (9.4%)	0.91	0.72–1.14	0.400				
BMI (median, range)	21.1 (13.1–36.0)	1.05	1.02–1.08	0.000				
≥30.0	18 (6.8%)							
≥35	2 (0.8%)							
<18.5	52 (27.2%)							
Osteoporosis	94 (35.3%)	1.15	0.89–1.49	0.270				
Diverticulosis	36 (13.5%)	1.27	0.89–1.82	0.190				
Morbus Crohn/Colitis ulcerosa	1 (0.4%)	0.35	0.05–2.51	0.300				
Cholecystolithiasis	13 (4.9%)	0.80	0.47–1.36	0.410				
Pre-transplant critical situation (e.g., MV, ECMO, ICU)	20 (7.5%)	0.68	0.42–1.09	0.110				
Pre-transplant ECMO	11 (4.1%)	0.64	0.33–1.36	0.180				
Lymphoma^a^(2pt)	2 (0.8%)	0.70	0.36–1.36	0.290				
Leukemia^a^(2pt)	0							
Tumor^a^(2pt)	11 (4.1%)	0.95	0.70–1.29	0.730				
Metastatic solid tumor^a^(6pt)	0							
AIDS^a^(6pt)	0							
^a^Charlson-Deyo-Index pt (median, range)	2 (1–6)	0.96	0.88–1.05	0.330				
1	76 (28.6%)				D	Ref		
2	99 (37.2%)					1.29	0.98–1.71	0.074
3	50 (18.8%)					1.04	0.72–1.49	0.840
4	20 (7.5%)					0.80	0.53–1.20	0.270
≥5	21 (7.9%)					1.28	0.95–1.72	0.100
Transplant and Donor Characteristics								
Era 1992–2000 vs. 2001–2019	50 (18.8%)	1.43	1.08–1.90	0.011	D	1.28	0.95–1.72	0.100
Era 1992–2008 vs. 2009–2019	146 (54.9%)	1.01	0.80–1.28	0.920				
Unilateral Transplantation	15 (5.6%)	0.71	0.41–1.23	0.220				
Re-Transplantation	7 (2.6%)	0.52	0.23–1.19	0.120				
Intra-operative ECMO use	126 (47.4%)	1.23	0.97–1.56	0.092				
CMV high risk	76 (28.6%)	1.27	0.98–1.66	0.075	A, B, C, D	1.32	1.01–1.74	0.026
Zurich Donor Score, median (range)	3 (0–11)	1.06	1.02–1.11	0.007	A, B, C, D	1.05	1.00–1.10	0.048
DCD	11 (4.1%)	0.95	0.51–1.77	0.880				
EVLP	4 (1.5%)	0.95	0.31–2.93	0.930				
PGD3 at T72	43/(16.2%)	1.19	0.84–1.68	0.340				

a Variables and points (pt) of Charlson-Deyo-Index.

Abbreviations: AIDS, acquired immune deficiency syndrome; BMI, body mass index; CI, confidence interval; CMV, cytomegalo virus; DCD, lung donation after circulatory death; HR, hazard ratio; MV, mechanical ventilation; ECMO, extracorporeal membrane oxygenation; EVLP, *ex vivo* lung perfusion; FEV1, forced expiratory volume in 1 s; ICU, intensive care unit; mPAP, mean pulmonary artery pressure; OR, odds ratio; PGD, primary graft dysfunction py, pack years.

### Definition of Comorbidities

The comorbidities in the CDI were defined by relying mostly on the original publication (4). In our selection program, all candidates with risk factors for coronary artery disease or aged ≥50 years old were evaluated by coronary angiogram. Congestive heart failure contains right or left heart failure or a combination of both. Right heart failure was defined as a mean pulmonary artery pressure (mPAP) >25 mmHg combined with echocardiographic evidence of right ventricular dysfunction (ventricular hypertrophy, moderate valve insufficiency, pericardial effusion) and/or signs of secondary liver or kidney dysfunction; left heart failure as having a reduced left ventricular ejection fraction <40%. Peripheral vascular disease includes aortic aneurysm, aortic ectasia and peripheral arterial disease grade I-IV. Cerebrovascular disease is defined as history of stroke with residual neurological deficit or transient ischemic attack. Connective tissue disease includes diagnosis of systemic lupus, rheumatoid arthritis, scleroderma, or seronegative spondyloarthropathy. Mild diabetes mellitus is type 1 and type 2 requiring medication, excluding dietary-controlled diabetes. For diabetes with end-organ damage renal, ophthalmic or neurological manifestations are required. Mild liver disease is defined as no portal hypertension with elevated liver enzymes more than three times the upper limit of normal. Moderate liver disease includes forms of fibrosis or cirrhosis causing portal hypertension with elevated liver enzymes. Moderate or severe renal disease includes glomerular filtration rate (eGFR) ≤60 ml/min/1.73 m^2^ or acute renal replacement therapy. Tumor means a history of malignancy, excluding non-melanoma skin cancer. Further comorbidities were selected based on the 2014 and 2021 ISHLT consensus statement (1) and availability. Thereby, systemic hypertension was defined as without treatment ≥140/90 mmHg; critical or unstable condition such as mechanical ventilation (MV), extracorporeal membrane oxygenation (ECMO) or other reasons requiring pre-operative ICU; and osteoporosis as bone density with T-score below −2.5. To screen for diverticulosis and other colon disorders, candidates ≥50 years of age (for cystic fibrosis ≥40 years) were evaluated by colonoscopy. Gastroscopy was performed in all candidates with history of gastrointestinal symptoms or age ≥50 years. Gastroesophageal reflux disease was diagnosed predominantly on symptoms or endoscopic or radiological evidence, rarely on manometry and pH-metry testing.

### Outcomes

The outcomes were PGD Grade-3 at 72 h, CLAD Grade-3 and survival after lung transplantation. PGD3-T72 is defined as PaO_2_/FiO_2_-ratio <200 mmHg and the presence of diffuse parenchymal infiltrates in the allograft on chest radiograph at 72 h after transplantation (9). As the definition was established in 2005, earlier cases were retrospectively analyzed by X-ray, ventilation curve and arterial blood gases. CLAD-3 is defined as a persistent decline of forced expiratory volume in 1 s (FEV1) ≤50% from baseline and an obstructive or restrictive physiology after exclusion of other causes (10).

### Definition of Donor and Era Variables

To consider the impact of donor factors, the Zurich-Donor-Score (11) was used. This score estimates the quality of donor lungs, based on 5 extended donor criteria: age, diabetes mellitus, smoking history, pulmonary infection, and ratio of partial pressure of arterial oxygen to inspired oxygen fraction. Due to change in induction and immunosuppression (Anti-thymocyte globuline to Basiliximab) therapy in 2000, this era effect was tested. Other arbitrary defined models splitting in two or three different eras of similar case size or years of transplant did not show any significant differences in survival.

### Statistical Methods

Statistical analysis was performed with IBM SPSS version 26 (SPSS IBM, Armonk, New York, USA) and R (Version 4.0.5, Vienna, Austria). Continuous data were compared using the Mann–Whitney test and categorical variables compared using the v^2^ test or the Fisher’s exact test for expected frequencies <5. Kaplan-Meier method was used to estimate survival as well as time to CLAD-3. The log-rank test compared survival curves. Cox regression was used to assess risk factors for mortality. Cox regression for CLAD-3 was adjusted for the competing factor of death by the Fine Gray methodology. Logistic regression was used to assess factors for PGD3-T72. First, every variable was checked with a univariate (enter) model. Variables with a *p-*value < 0.2 (12) were tested in a multivariate stepwise backward Cox regression model or linear regression model, respectively. The number of factors introduced into the final multivariable model was calculated by considering sample size and number of occurring events (13). To confirm that variables show a stable significance, they had to be frequent in number. Linear regression was used to test collinearity between variables. A variance inflation factor >5 and a tolerance <0.2 was defined as indicating a collinearity problem. Different final multivariate models are provided to bypass variables with statistical or clinical collinearity. In general, a *p*-value < 0.05 was considered to be the threshold for statistical significance.

The local research ethics review committee approved the study (KEK-Nr.2019-00873).

## Results

**FIGURE 1:**
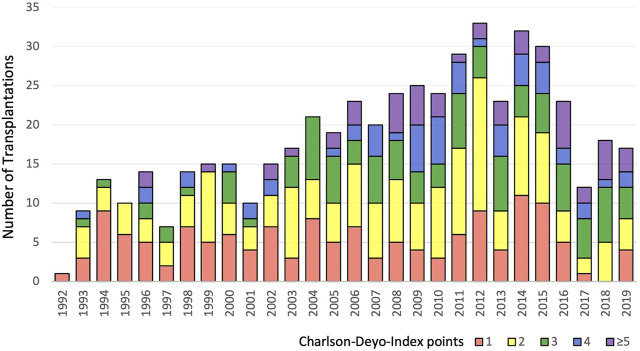
Distribution of the recipient comorbidity burden over the study period, measured by the Charlson-Deyo-Index. The first scoring point accounts for the always present underlying lung disease.

In our study population, there were 513 adult recipients who underwent lung transplantation between 1992 and 2019. Of these, 353 recipients (68.8%) died, 266 (51.9%) developed CLAD-3 and 79 (15.4%) PGD3-T72. Median follow-up time was 12.7 years. No loss to follow-up occurred. Half of the transplants were performed in the era 1992–2008 and showed a trend of better survival than the era 2009–2019 (median survival 8.4 vs. 5.9 years, respectively, log-rank = 0.092). The same was observed for onset of CLAD-3 (median 7.6 vs. 5.5 years, respectively, log-rank = 0.121). In line with these trends, donor marginality measured by ZDS (mean 2.8 vs. 4.0 points, *p* < 0.001) and the recipient comorbidity burden measured by CDI (mean 2.2 vs. 2.7 points, *p* < 0.001) increased significantly in the second era. Figure 1 shows the detailed increase of the CDI score burden over the study period. In the earlier era a trend of more PGD3-T72 occurred (17.8% vs. 13.2%, respectively, *p* = 0.144).

**FIGURE 2:**
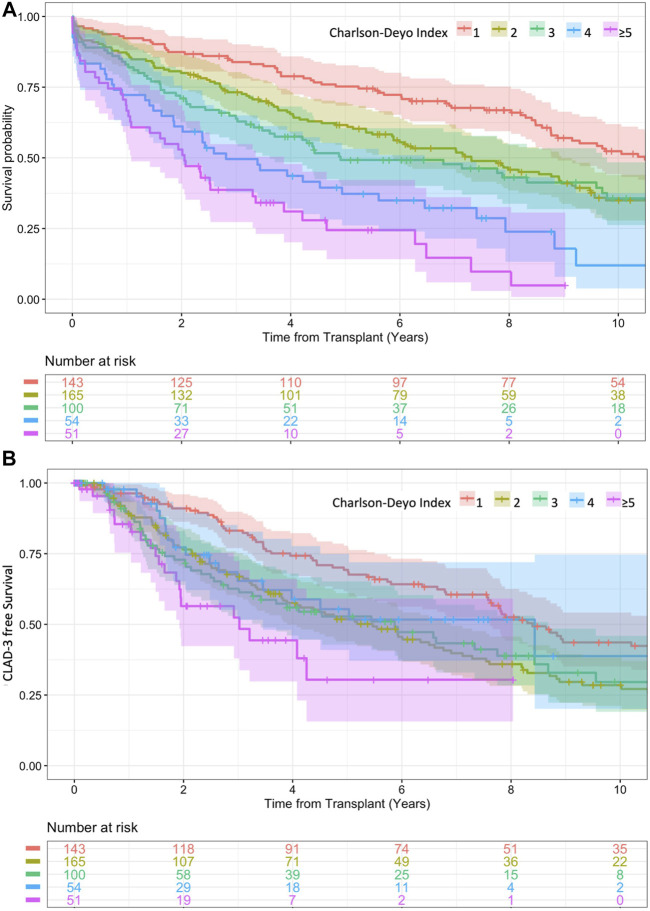
**(A)** Kaplan-Meier survival curves for different comorbidity burdens in the Charlson-Deyo-Index. 1 vs. 2 log-rank = 0.004, 2 vs. 3 log-rank = 0.776, 3 vs. 4 log rank = 0.020, 4 vs. ≥5 log rank = 0.045. **(B)** Kaplan-Meier curve for onset of CLAD-3 for different comorbidity burdens in the Charlson-Deyo-Index. 1 vs. 2 log-rank = 0.001, 2 vs. 3 log-rank = 0.927, 3 vs. 4 log rank = 0.537, 4 vs. ≥5 log rank = 0.059.

Seventy two percent of the recipients had at least one comorbidity represented in the CDI, beside of the always present underlying chronic pulmonary disease which accounts for an extra point. As illustrated in the Kaplan-Meier survival curve of Figure 2A, an increasing number or severity of comorbidities in the CDI was associated with significantly poorer survival, except that a score of 2 points was comparable to a score of 3 points (log-rank = 0.776). The median survival for a CDI score of 1, 2, 3, 4 and ≥5 points was 10.5, 7.3, 4.9, 2.8, and 2.1 years, respectively.

For the overall population, detailed descriptive statistics of recipient-, donor-, intra-operative characteristics are shown in Table 1. The most frequent underlining diseases were cystic fibrosis (30%) and emphysema (30%). The most frequent comorbidity was congestive heart failure (52%) including in 98% of these cases right heart failure all with an mPAP >25 mmHg. The next most frequent comorbidities were osteoporosis (35%), gastroesophageal reflux (29%), systemic hypertension (27%), mild diabetes (18%), mild liver disease (15%), diverticulosis (13%) and moderate to severe renal disease (12%).

### Risk Factors for Survival

All comorbidities listed in Table 1 were assessed in univariable and if applicable in multivariable risk analysis. In multivariable Cox regression (Table 1, Model A), moderate liver disease, peptic ulcer disease, gastroesophageal reflux, diabetes with end-organ damage, moderate to severe renal disease, osteoporosis, diverticulosis, and congestive heart failure were independent risk factors for mortality, beside of increasing age, increasing ZDS and unilateral lung transplantation. The subgroups of left heart failure and right heart failure as well as mPAP >25 mmHg, chronic atrial fibrillation and systemic hypertension were also multivariate risk factors for mortality when independently analyzed from congestive heart failure (Table 1, Model B, C). Of note, the underlying lung diseases were no multivariable risk factors in the models, after introducing comorbidities. The same effect was found for re-transplantation, pre-transplant critical situation, ECMO as bridge to transplantation and intraoperative ECMO use.

The accumulation of comorbidities with CDI in the multivariable model (Table 1, Model D) showed an even better performance for survival estimates than the unadjusted Kaplan-Meier curves (Figure 2A).

### Risk Factors for PGD3-T72

Recipient-, donor-, intra-operative characteristics for those transplantations where PGD3-T72 occurred are listed in Table 2. In this subpopulation, the underlying diagnosis of idiopathic pulmonary fibrosis (35%, *p* = 0.001) and idiopathic pulmonary arterial hypertension (18%, *p* < 0.001) were significantly higher represented. The percentage of congestive heart failure (81%, *p* < 0.001), a mPAP >25 mmHg (79%, *p* < 0.001), ECMO as bridge to transplantation (13%, *p* = 0.019), intraoperative ECMO use (76%, *p* < 0.001), CDI (*p* = 0.006) and ZDS (*p* = 0.011) were also significantly higher than in the overall population.

In multivariable logistic regression congestive heart failure, a BMI>30kg/m2, an increasing ZDS and intraoperative ECMO use were independent risk factors for PGD3-T72 (Table 2, Model A). Additional analyses excluding congestive heart failure revealed, that mPAP >25 mmHg and right heart failure were also factors for PGD3-T72 (Table 2, Model B, C). The accumulation of comorbidities in the CDI was associated with the risk of PGD3-T72 but not in a linear increasing way with increasing scoring points (Table 2, Model D), likely due to the small sample size.

### Risk Factors for Onset of CLAD-3

For the subpopulation of CLAD-3, recipient-, donor-, intra-operative characteristics are listed in Table 3. The CLAD-3 subpopulation was comparable to the overall population with respect to the underlying disease and variables of intraoperative procedure, but showed a trend to more marginal donor lungs in the ZDS (*p* = 0.097) and a significantly higher comorbidity burden in the CDI (*p* = 0.018).

Multivariate Cox regression revealed that the underling diagnosis of idiopathic pulmonary fibrosis, a smoking history of the recipient of >20 packyears, epilepsy, CMV high-risk constellation and an increasing ZDS were independent risk factors for onset of CLAD-3 (Table 3, Model A, B, C). Congestive heart failure, right heart failure and mPAP >25 mmHg were borderline risk factors (Table 3, Model A, B, C). The change in induction and immunosuppression in 2000 from Anti-thymocyte globuline to Basiliximab was a borderline risk factor (Table 3, Model D). Recipient age and PGD-3 were no risk factors for developing CLAD-3.

Moreover, the comorbidity burden estimated by CDI was not a multivariable risk factor for developing CLAD-3 (Table 3, Model D). This is in line with the Kaplan-Meier estimate, where onset of CLAD-3 was not gradually reduced by an increasing CDI (Figure 2B). The median time until onset of CLAD-3 for a CDI score of 1, 2, 3, 4 and ≥5 points was 8.4, 5.5, 5.9, 8.4, and 3.0 years, respectively.

## Discussion

This study is the first detailed analysis of association between recipient comorbidities prior to transplantation and survival, PGD3-T72 and onset of CLAD-3 after lung transplantation. We show that several recipient comorbidities and their accumulation have a strong impact on post-transplant survival, and that some comorbidities also affect the development of PGD3-T72 and CLAD-3.

It is paramount to define the right time of listing and transplanting a candidate. On one hand, a limited life expectancy due to the lung disease is required to justifying the benefit over the risk of a lung transplantation. On the other hand, a prolonged time span until transplantation is often associated with developing a more extensive comorbidity profile. This problem is further aggravated by a demographic shift toward older candidates, who are per se more likely to be multi-morbid.

While lung transplantation may improve previously poor organ oxygenation and consecutively slow down the progression of many comorbidities, surgical complications and the side effects of the immunosuppression regime may worsen comorbidities considerably and even create new comorbidities over time.

In addition to respecting the ISHLT consensus document (1) for absolute contraindications, our center has been fairly liberal in the acceptance of candidates with reasonable comorbidities. Estimated by the CDI, 72% of our recipients had at least one comorbidity in addition to the underlying lung disease, providing ideal conditions for a thorough analysis.

### Factors Associated With Survival

Among pretransplant recipient comorbidities, we identified right heart failure as an important risk factor affecting survival, PGD3-T72 and partially also CLAD-3. It was the most frequent comorbidity found in half of our cohort. Right heart failure and especially its approximative surrogate of pulmonary hypertension >25 mmHg were also risk factors for mortality in a single center study (14) and in the United Network for Organ Sharing (UNOS) Database in 3105 emphysema patients (15). Even though right heart failure may be partially to fully reversible after lung transplantation, pulmonary hypertension requires sometimes peri-operative extracorporeal membrane oxygenation (ECMO) implantation to avoid reperfusion edema which goes along with a variety of factors that can increase morbidity (16). One of the morbidities is PGD attributed to the systemic inflammatory response associated with the machine as well as its systemic anticoagulation requirements (17). In our cohort, intraoperative ECMO use was also an independent risk factor for developing PGD3-T72.

In our study, the few cases of left heart failure were also strongly associated with mortality. Previous reports about left heart failure are lacking, likely as it is widely considered a contraindication for transplantation (2).

Systemic hypertension was present in one fourth of our cohort. It was a risk factor for mortality, in line with a previous report in 821 pulmonary fibrosis recipients (18). Pretransplant systemic hypertension may aggravate differently after transplant because of the side effects of immunosuppression treatment with calcineurin inhibitors than in previously non-hypertensive recipients. This might lead to earlier end organ damage. Moreover, a meta-analysis (19) has shown that systemic hypertension was a risk factor for postoperative atrial arrhythmias and therefore had prognostic implications for length of hospital stay and overall survival.

Pretransplant atrial fibrillation increased the risk of adverse cardiovascular outcomes and longer hospital stay in a single-center study (20). In our study, pretransplant chronic atrial fibrillation was even an independent risk factor for mortality.

We identified diabetes mellitus with end-organ damage but not mild diabetes as a risk factor for mortality. This is in line with the findings of the University of Melbourne study (21) for poorly controlled glycemic controlled candidates. The ISHLT report even lists any stage of diabetes as a risk factor for 10-year mortality (22), including diabetes without end organ damage.

Renal disease may further aggravate in the peritransplant period mainly due to the immunosuppression regimen and fluid shifts after transplantation. Moderate to severe renal disease was an independent risk factor for mortality in our cohort. An eGFR of 60 ml/min/1.73m2 or less was also an independent risk factor for 1-year survival using UNOS data (23). And the ISHLT report lists recipient with a pre-transplant dialysis condition as a risk factor for 10-year mortality (22).

Currently, the impact of moderate liver disease is poorly understood because it has hardly been investigated so far. Although we found moderate liver disease to be a risk factor for mortality in our cohort, liver cirrhosis with or without portal hypertension did not have a negative impact on 5-year survival in 6 matched cystic fibrosis recipients in a previous study (24).

Gastroesophageal reflux was suggested to be associated with secondary aspiration contributing to acute rejection, pulmonary infection and CLAD and consecutive mortality (25). However, even though gastroesophageal reflux was an independent risk factor for mortality in our cohort, no risk association was found for development of PGD3-T72 and CLAD-3. A reason might be that several asymptomatic recipients were insufficiently screened in our program (26), preventing a correlation to PGD and CLAD. Another reason may be that we universally teach patients about anti-reflux measures (27).

Peptic ulcer disease was also a risk for mortality in our study. It is reported from small series to occur and reoccur after transplantation and may lead to intestinal perforation (28, 29).

The rate of developing acute diverticulitis from preexisting diverticulosis in immunosuppressed patients is significantly higher than in the general population (30). At our center, we reported an overall rate of diverticulitis of 4.5% after lung transplantation (31).

The prevalence of osteoporosis affected one third of our cohort and it was a significant risk factor for survival. Osteoporosis is in part reflected by preoperative steroid use which was a risk factor for 1-year survival in a study using UNOS data (23).

Neither mild nor post-interventional coronary disease were independent risk factors in our cohort, which is in line with previous studies (32, 33). Our cases with a history of myocardial infarction might have been too few in number or too highly selected to become an independent risk.

Multiple reports on other solid organ transplantations indicate that the presence of symptomatic peripheral vascular disease is one of the strongest predictors of mortality (34-36). In our study, a mild peripheral artery disease grade I seems to have minor impact on post-transplant survival. Previous aortic dissection and aortic ectasia appeared to be associated with post-transplant mortality in univariable analysis, but the limited number in our cohort did not justify further analysis.

We noted, that the underlying lung disease, a preoperative critical situation, and re-transplantation lost their strength as risk factors for mortality, when analyzed along with comorbidities. These variables may consecutively be regarded as surrogates for the comorbidity burden. For an optimal candidate selection, the focus should therefore lie on the comorbidity profile.

### Factors Mainly Associated With PGD3-T72

In addition to right heart failure and mPAP >25 mmHg, described above, a BMI>30 kg/m^2^ was a strong risk factor for developing PGD3-T72 in our study. Pulmonary hypertension and BMI >25 were also reported as independent risk factors for PGD in a cohort of 7322 recipients (37) and in a meta-analysis (38). The mechanism of adipositas on PGD is not yet fully understood. It is likely caused by comorbidities associated with adipositas. This would also explain why adipositas was not a multivariable risk factor for mortality in our study.

### Factors Mainly Associated With CLAD-3

This study is the first to detect epilepsy as a risk factor for CLAD-3. Some anti-epileptic medication show side effects on respiratory depression, increase oral and pulmonary secretions and even interstitial lung disease (39). Moreover, epilepsy might go along with an increased risk of aspiration leading to pneumonia, inflammation, and consecutive fibrotic alterations of the lung allograft. An additional risk for CLAD-3 was a previous smoking history of more than 20 packyears. We do not believe that the systemic damages caused by previous smoking is responsible for this effect, but the increased likelihood of being still exposed to a smoking environment or even due to smoking resumption (40). Another important aspect is the underlying disease in particular idiopathic pulmonary fibrosis. It was an independent factor for developing CLAD. The process may be due to the re-occurrence of the underlying disease in the allograft.

PGD was repeatedly associated with the risk of developing CLAD (41). However, we could not find such a correlation in our cohort. The detected borderline risk of a pre-transplant mPAP >25 mmHg might occasionally have caused *de novo* pulmonary hypertension and chronic lung edema and fibrosis of the lung allograft.

### Charlson-Deyo-Index

An increasing comorbidity burden, estimated by the CDI, was well associated with an increasing risk for mortality. We showed that already one proportionally mild comorbidity in the CDI bears a significant risk on survival outcome. This should emphasize that a very careful selection of candidates considering comorbidities is crucial. However, we can not provide a recommendation based on our single-center analysis.

Our finding of CDI as a good predictor for survival is in line with multiple studies of other solid organ transplants (5-8). However, the Pittsburgh group (42) calculated the original Charlson Index for 748 lung transplant recipients and neither detected an association with in-hospital post-transplant complications nor an association with survival in a multivariate model. This might be due to an incomplete assessment of comorbidities, incomplete adjustment for confounders, and incorporation of recipient age in the score.

We detected several other comorbidities beyond the 18 comorbidity conditions represented in the CDI as important risk factors for survival. Thus, the addition of other comorbidities, a different weighing or sub-categorization may even improve the prediction of the CDI in the context of lung transplantation. This would have to be determined and proven in future studies.

The association of the comorbidity burden in the CDI was weaker for PGD3-T72 than for survival. Only one comorbidity of the CDI, congestive heart failure, was independently associated with onset of PGD3-T72 and borderline associated with onset of CLAD-3. Mechanisms of developing PGD and especially CLAD appear to rely more on a limited number of specific comorbidities, rather than on their quantity.

### Limitations

This study has several limitations. It is a retrospective single-center study over more than 2 decades. The pre- and posttransplant treatment of some comorbidities might have changed over time. However, we could not detect an era effect in univariable and multivariable analyses. Some comorbidities might have been underrepresented in our study, which would have otherwise been important risk factors.

## Conclusion

Our study identified several comorbidities that were associated with post-transplant survival, onset of PGD and CLAD. Based on our findings we consider the comorbidities mentioned in the current ISHLT-consensus document (2) as relative contraindications as valid risk factors for mortality after lung transplantation. The CDI may potentially be used for a more refined evaluation of multimorbid candidates.

## Data Availability

The datasets presented in this article are not readily available because Potential conflict with the Swiss Privacy Act, a federal law. The data that support the findings of this study are only available from the corresponding author, upon reasonable request. Requests to access the datasets should be directed to II, ilhan.inci@usz.ch.

## Abbreviations

CDI, Charlson-Deyo-Index; CLAD, chronic lung allograft dysfunction; ISHLT, International Society for Heart and Lung Transplantation; mPAP, mean pulmonary artery pressure; UNOS, United Network for Organ Sharing; ZDS, Zurich Donor Score.
